# NTPDases in the neuroendocrine hypothalamus: Possible energy regulators of the positive gonadotrophin feedback

**DOI:** 10.1186/1477-7827-7-63

**Published:** 2009-06-16

**Authors:** Attila Zsarnovszky, Tibor Bartha, Laszlo V Frenyo, Sabrina Diano

**Affiliations:** 1 Department of Physiology and Biochemistry, Szent Istvan University Faculty of Veterinary Sciences, Budapest, Hungary; 2 Department of Obstetrics, Gynecology & Reproductive Sciences, Yale University School of Medicine, New Haven, Connecticut 06510, USA; 3 Department of Neurobiology, Yale University School of Medicine, New Haven, Connecticut 06510, USA

## Abstract

**Background:**

Brain-derived ectonucleoside triphosphate diphosphohydrolases (NTPDases) have been known as plasma membrane-incorporated enzymes with their ATP-hydrolyzing domain outside of the cell. As such, these enzymes are thought to regulate purinergic intercellular signaling by hydrolyzing ATP to ADP-AMP, thus regulating the availability of specific ligands for various P2X and P2Y purinergic receptors. The role of NTPDases in the central nervous system is little understood. The two major reasons are the insufficient knowledge of the precise localization of these enzymes in neural structures, and the lack of specific inhibitors for the various NTPDases. To fill these gaps, we recently studied the presence of neuron-specific NTPDase3 in the mitochondria of hypothalamic excitatory neurons by morphological and functional methods. Results from those studies suggested that intramitochondrial regulation of ATP levels may play a permissive role in the neural regulation of physiological functions by tuning the level of ATP-carried energy that is needed for neuronal functions, such as neurotransmission and/or intracellular signaling.

**Presentation of the hypothesis:**

In the lack of highly specific inhibitors, the determination of the precise function and role of NTPDases is hardly feasable. Yet, here we attempt to find an approach to investigate a possible role for hypothalamic NTPDase3 in the initiation of the midcycle luteinizing hormone (LH) surge, as such a biological role was implied by our recent findings. Here we hypothesize that NTPDase-activity in neurons of the AN may play a permissive role in the regulation of the estrogen-induced pituitary LH-surge.

**Testing the hypothesis:**

We propose to test our hypothesis on ovariectomized rats, by stereotaxically injecting 17beta-estradiol and/or an NTPDase-inhibitor into the arcuate nucleus and determine the consequential levels of blood LH, mitochondrial respiration rates from arcuate nucleus synaptosomal preparations, NTPDase3-expression from arcuate nucleus tissue samples, all compared to sham and intact controls.

**Implications of the hypothesis:**

Results from these studies may lead to the conclusion that estrogen may modulate the activity of mitochondrial, synapse-linked NTPDase3, and may show a correlation between mitochondrial NTPDase3-activity and the regulation of LH-release by estrogen.

## Background

NTPDases (also known as ectonucleotidases or ecto-apyrases) have been described as an 8-member family of nucleotidase enzymes. Most NTPDases are integral membrane proteins: NTPDase1 [1], NTPDase2 [2], NTPDase3 [3,4] and NTPDase8 [5] reside in the plasma membrane with their active site outside of the cell. These cell surface enzymes hydrolyze extracellular nucleotides, thus regulating the availability of specific ligands for P_2_X and P_2_Y purinergic receptors. In certain tissues, nucleoside monophosphates are further hydrolyzed by 5'-ectonucleotidase to adenosine that activates P_1 _adenosine receptors. NTPDase4–8 have not been detected in the brain.

Of the NTPDases, types 1, 2 and 3 have been identified and studied in the brain. NTPDase1 was identified in neurons, glia and endothelial cells of the rat brain [6,7], whereas NTPDase2 was detected mostly in the germinal zones of the rat CNS; Type-B cells also expressed NTPDase2 [8]. NTPDase3 mRNA was first identified in the brain by Chadwick and Frischauf [4], and we recently determined the tissue distribution of this enzyme in the rat brain [9]. In the latter study, we suggested that NTPDase3 is neuron-specific, with the enzyme being present in neuronal perikarya and neuronal processes. NTPDase3-immunoreactive (NTPDase3-IR) perikarya were only observed in the arcuate nucleus (AN) and the lateral hypothalamic nucleus (LHN). In our most recent work we further characterized the subcellular localization of NTPDase3 in the hypothalamus [10]. As part of the latter correlated light- and electron microscopic examinations, we identified NTPDase3-IR in the mitochondrial matrix or closely linked to the inner mitochondrial membrane of hypothalamic neurons. Additionally, immunohistochemical and electron microscopic studies strongly implied that hypothalamic NTPDase3-IR may only be found in excitatory neurons. Those morphological results demonstrating NTPDase3 in the neuronal mitochondrial matrix were confirmed by functional studies where synaptosomal fractions from hypothalamic tissue homogenates were subjected to mitochondrial respiration measurements. We found that decrease of NTPDase-activity by the use of an NTPDase- (including NTPDase3) inhibitor resulted in significantly decreased ADP-dependent state 3 mitochondrial respiration rate and total mitochondrial respiratory capacity. Since neuronal activity, especially neurotransmission is highly energy dependent [11], it was reasonable to assume that hypothalamic neuronal activity, especially that of excitatory neurons, may be dependent on the activity of mitochondrial NTPDase3 due to the ATPase activity of this enzyme.

The neuroendocrine hypothalamus is the target of a number of peripheral hormones that function as signals for the feedback-based regulation of various homeostatic systems. Estrogen, for example, targets numerous hypothalamic neurons, including those of the arcuate nucleus, to regulate the GnRH- and consequential pituitary LH-surge. We and others have previously demonstrated that in non-human primates, as well as in rats, considerable synaptic events take place in the neuroendocrine hypothalamus, termed the „estrogen-induced hypothalamic synaptic plasticity“ (EISP). EISP is marked by a well-defined pattern of synaptic reorganization of both excitatory (mainly during the positive gonadotrophin feedback) and subsequently, inhibitory interneuronal contacts [12,13]. Since the AN (besides the LHN) is one of the hypothalamic structures hosting NTPDase3-IR neuronal perikarya, we wanted to know whether varying estrogen levels influence the hypothalamic expression level of NTPDase3. Therefore, we carried out studies [10] to investigate the effects of the absence or presence of estrogen on medial- and lateral hypothalamic NTPDase3-levels. Results from Western blot analyses showed a time- and estrogen-dependent pattern of changes in NTPDase3-expression levels in both the medial and lateral sides of the sampled hypothalami, interestingly however, the temporal pattern of changes in the medial and lateral sides differed. Specifically, in samples including the LHN, NTPDase3 levels increased significantly 4–12 hrs after a single subcutaneous injection of estrogen, and gradually returned to nearly control (ovx) levels by 16–26 hrs after estrogen-treatment. In contrast, temporal changes in medial hypothalamic samples including the AN showed an initial increase in NTPDase3-expression between 6–10 hrs after estrogen-treatment, followed by a sharp decrease to control levels, and again followed by a second rise between 22–26 hrs after estrogen-treatment. Thus, in the lateral hypothalamus a single-, whereas in the medial hypothalamus a double-peaked curve was determined, suggesting that NTPDase3-expression in the hypothalamus is regulated by estrogen, however, the role of these enzymes (including NTPDase3) in the two hypothalamic regions might be different. Based on the aforementioned double-peaked curve detected in medial hypothalamic samples, it was reasonable to assume that the temporal changes in medial hypothalamic NTPDase3-expression could correspond to the initiation of the two successive phases of the GnRH-regulating feedback mechanism, i.e., the estrogen-induced positive- and successive negative (reciprocal) feedbacks. Our ongoing pilot experiments also show that 10 hours after estrogen-treatment, when hypothalamic NTPDase3 levels are high, inhibition of NTPDases in synaptosomes from medial hypothalamic samples results in a more robust fall in oxygen consumption during state 3 mitochondrial respiration, suggesting that estrogen-treatment increased NTPDase-activity in synaptosomal mitochondria. It is important to note that previous studies carried out on synaptosomes from several brain regions suggested that the ecto-ATPase activity detectable under such experimental conditions is most likely the result of NTPDase3-, rather than any of the two other (NTPDase1 or NTPDase2) NTPDases present in the CNS [14,15]. Additionally, in a review summary by Langer et al. [16] of the present knowledge of the distribution of different NTPDases in the brain, the authors arrived to the conclusion that in the lack of considerable amounts of other NTPDases, much of the ATPase activity in the hypothalamus is attributable to NTPDase3. These observations argue for a role of NTPDase3 in the tuning of neuronal ATP-availability in the hypothalamus and, consequentially, for a permissive role of NTPDase3 in the initiation of EISP and in the regulation of the pituitary LH-release.

### Presentation of the hypothesis

Our initial results described above lead us to the hypothesis that NTPDase-activity in neurons of the AN may play a permissive role in the regulation of the estrogen-induced pituitary LH-surge.

### Testing the hypothesis

At least five healthy, normal cycling female rats per each parameter examined (see below) will be used for the study. Animals will be ovariectomized (ovx) 7 days prior to further experimentation. Seven days after ovariectomy, animals will receive stereotaxic intracerebral (i.c) injections into the arcuate nucleus, using the stereotaxic coordinates provided by Paxinos and Watson [17], as follows: Group 1 will receive 17beta-estradiol (E_2_) 20 minutes after initial injection of saline; Group 2 will receive E_2 _20 minutes after initial i.c. injection of suramin (NTPDase-inhibitor); Group 3 will receive two consecutive injections of physiological saline (sham control) with a timing presented above. An additional group of animals (Group 4) not subjected to i.c. injections will be investigated as described below. Animals will be sacrificed 2-6-10-18 and 26 hours after the i.c. injections. Blood samples will be drawn from each individual and plasma LH will be determined. Brains will be quickly removed and tissue samples containing the arcuate nucleus will be obtained by punch sampling biopsy technique as described by Palkovits and Brownstein [18]. Tissue samples will be homogenized and protein concentrations quickly determined. Based on sample protein concentrations, aliquotes will be prepared from tissue homogenates for further determination of NTPDase3-levels by Western blot analysis.

Mitochondrial oxygen consumption (mitochondrial respiration rates, especially ADP-dependent state 3 respiration and total mitochondrial respiratory capacity) will be determined from synaptosomal preparations of tissue aliquotes without further inhibition (suramin) of the sample (i.e., the left-sided medial hypothalamus), or with further suramin-treatment of the contralateral (i.e., the right-sided) sample. Please note that the initial, i.c. injection of suramin is necessary to test the effect of NTPDase-inhibition on plasma LH levels (mitochondrial respiration measurement results of left-sided samples should be viewed in light of the LH concentration measured), while further *in loco *treatment of the right-sided samples allows the determination and therefore the better comparison of NTPDase activities among group-members. It should be noted that since no selective NTPDase3-inhibitor is available to-date, the proposed experiments will also be performed using alternative inhibitors that are preferably less potent purinoceptor antagonists. It is also necessary that the results from the proposed experiments be confirmed once a specific inhibitor, that does not affect purinergic receptors but is highly specific for NTPDase3, becomes available.

**Figure 1:**
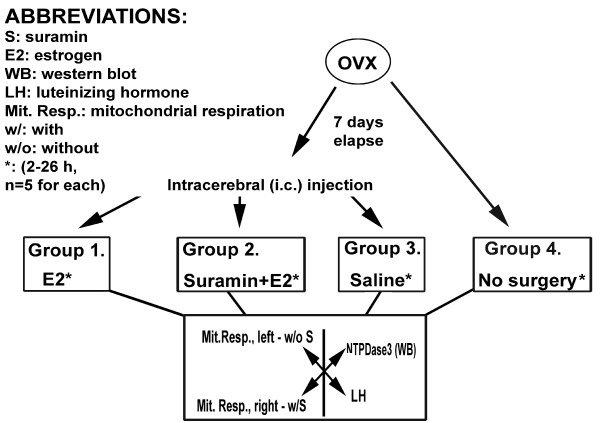
**Experimental design for testing the proposed hypothesis**. Animals will be ovariectomized (ovx) 7 days prior to further experimentation. Seven days after ovariectomy, animals will receive intracerebral (i.c.) stereotaxic injections into the arcuate nucleus as follows: Group 1 will receive 17beta-estradiol (E_2_) 20 minutes after initial injection of saline; Group 2 will receive E_2 _20 minutes after initial i.c. injection of suramin (NTPDase-inhibitor); Group 3 will receive two consecutive injections of physiological saline (sham control) with a timing presented above. An additional group of animals (Group 4) not subjected to i.c. injections will be investigated. Animals will be sacrificed 2-6-10-18 and 26 hours after the i.c. injections. Blood samples will be drawn from each individual and plasma LH will be determined. Brains will be quickly removed and the medial part of the hypothalami will be isolated. Tissue samples from left and right sides will be separately handled. Tissue samples will be homogenized and protein concentrations quickly determined. Based on sample protein concentrations, aliquotes will be prepared from tissue homogenates for further determination of NTPDase3-levels by Western blot analysis. Mitochondrial oxygen consumption (mitochondrial respiration rates, especially ADP-dependent state 3 respiration and total mitochondrial respiratory capacity) will be determined from synaptosomal preparations of tissue aliquotes without further inhibition (suramin) of the sample (i.e., the left-sided medial hypothalamus), or with further suramin-pretreatment of the contralateral (i.e., the right-sided) sample.

The experimental design is summarized in Figure 1.

### Implications of the hypothesis

With the proposed hypothesis it is our goal to prompt further studies that investigate the relationship between the NTPDase-dependent energetic status of cells and their relevant biological functions.

NTPDase3 has been previously found in the mediobasal hypothalamus [9]. Since one of the major roles of the AN is to regulate the GnRH and consequential LH release, the presence of NTPDase3 in AN neurons implied a role for this enzyme in the aforementioned hypothalamic regulatory mechanism. Since, however, NTPDase3 has been generally known as a trans-membrane enzyme that hydrolyzes ATP to modulate the availability of specific ligands for various purinergic receptors, one would speculate that such biological role, if exists, for NTPDase3 is achieved by the influence of NTPDase3 enzymatic activity on purinergic neurotransmission. This idea would be supported by a number of previous observations, including and involving the function of various purinoceptors, [19] in the mediobasal hypothalamus. It is conflicting, however, that no evidence surfaced to-date that would indicate a role for purinoceptors in the AN in the regulation of GnRH/LH release. Therefore, based on our previous findings [10], it is reasonable to assume that NTPDase3 may influence GnRH/LH release through intracellular enzymatic activity, one of which may involve the mitochondrial regulation of ATP amounts. To-date, NTPDase3 has been found in several organs including the CNS, pancreas, cardiomyocytes, lung respiratory epithelium and the Leidig cells of the testis; and in each case, NTPDase3-function was considered as an extracellular ectonucleotidase activity functionally linked to purinergic signaling. In other words, the role of NTPDase3 in the listed organ- or tissue specific functions was, therefore, discussed in the context of a membrane-bound ATPase-ADPase enzyme. Unfortunately, those studies failed to investigate and determine the subcellular localization of NTPDase3 by any means, including electron microscopy or subcellular fractionation. Therefore, the idea of subcellular, compartment-linked NTPDase functions in the CNS has not emerged.

While the function of NTPDase3 has been mostly tested by receptor antagonists, increasing efforts have been made to find selective blockers for the enzyme itself. Despite of those efforts, a fully specific inhibitor has not yet been found. Today, the lack of such specific blockers is a considerable drawback of NTPDase-research. Yet, some of the purin receptor antagonists are also capable of inhibiting NTPDase enzyme activity. One such inhibitor for NTPDases is the polyoxometalate suramine. Although suramine (and its analogues) cannot be considered for selective inhibition of receptors or NTPDase-activity, it can be used in some subcellular fractions of biological samples, such as synaptosomal fractions isolated from various sources. As cited above, few studies investigated the ATPase activity of brain NTPDases in synaptosomal fractions, however, none of those examined a functional link between mitochondrial respiration and NTPDase activity. We, in our most recent study, demonstrated that inhibition of NTPDase activity in synaptosomal fractions decreased ADP-dependent state 3 mitochondrial respiration, and that the hypothalamic NTPDase3 expression and the mitochondrial state 3 respiration rates are dependent on the actual estrogenic background [10]. These results suggest the possibility that estrogen may set the amount of mitochondrial ATP near synapses and thereby influence synaptic transmission by modulating its ATP-supply.

### Expected results

As described in the hypothesis, we expect that inhibition of NTPDases in the AN would prevent the estrogen-induced pituitary LH release. Inhibition of AN NTPDase-activity may affect changes in intercellular purinergic signaling and may also influence intracellular energy states by the hydrolytic activity of mitochondrial NTPDase.

Numerous studies reported that extracellular ATP increases the activity level of GnRH neurons and the pituitary gonadotrops [20-23]. On this line one would speculate that the inhibition of extracellular ATP hydrolysis by NTPDases would result in longer-lasting elevated extracellular ATP levels leading to the facilitation of the pituitary LH-release. However, to the best of our knowledge, no evidence exists that would suggest a role for AN purinoceptors in the regulation of GnRH/LH release. Therefore, by the local, intra-AN application of suramin, the effects of the inhibitor on neurons outside of the AN (such as the medial preoptic area and the anterior pituitary) could be discounted, and the effects could be attributed to the intracellular inhibition of NTPDases, NTPDase3 in particular.

The proposed, yet to be tested mechanism would involve the inhibition of mitochondrial NTPDase-activity in hypothalamic neurons, thereby leading to a momentary increase in mitochondrial ATP levels, but a subsequent silencing of mitochondrial respiration (as suggested by the decrease of state 3 mitochondrial respiration rates after NTPDase-inhibition); As a result, a halt in mitochondrial ATP production would occur. In this case, NTPDase-containing nerve terminals would not have sufficient amounts of ATP to fuel neurotransmission, therefore, the highly energy-demanding EISP could not be initiated, thus preventing pituitary LH-release.

In any case, testing the possible influence of NTPDase-inhibition on pituitary LH-release will bring us closer to understand the relationship between the considerably versatile neuronal ATP functions and the hypothalamic regulation of pituitary LH-release.

## Competing interests

The authors declare that they have no competing interests.

## Authors' contributions

The authors, through their frequent and periodic scientific consultations, have taken an equal share in the formulation of the presented hypothesis article. All authors read and approved the final manuscript.
